# Efficacy of antiviral therapy and host–virus interactions visualised using serial liver sampling with fine-needle aspirates

**DOI:** 10.1016/j.jhepr.2023.100817

**Published:** 2023-06-19

**Authors:** Samuel C. Kim, Jeffrey J. Wallin, Yanal Ghosheh, Muhammad Atif Zahoor, Juan Diego Sanchez Vasquez, Shirin Nkongolo, Scott Fung, Patricia Mendez, Jordan J. Feld, Harry L.A. Janssen, Adam J. Gehring

**Affiliations:** 1 Gilead Sciences, Inc., Foster City, CA, USA; 2 Toronto Centre for Liver Disease, Toronto General Hospital Research Institute, University Health Network, Toronto, ON, Canada; 3 Department of Immunology, University of Toronto, Toronto, ON, Canada; 4 Department of Internal Medicine IV (Gastroenterology, Hepatology, Infectious Diseases), University Hospital Heidelberg, Heidelberg, Germany; 5 Erasmus Medical Center, Division of Gastroenterology and Hepatology, Rotterdam, The Netherlands

**Keywords:** Hepatitis B, Hepatocyte, Viral-Track, FNA, Fine needle aspirate, Liver

## Abstract

**Background & Aims:**

Novel therapies for chronic hepatitis B (CHB), such as RNA interference, target all viral RNAs for degradation, whereas nucleoside analogues are thought to block reverse transcription with minimal impact on viral transcripts. However, limitations in technology and sampling frequency have been obstacles to measuring actual changes in HBV transcription in the liver of patients starting therapy.

**Methods:**

We used elective liver sampling with fine-needle aspirates (FNAs) to investigate the impact of treatment on viral replication in patients with CHB. Liver FNAs were collected from patients with CHB at baseline and 12 and 24 weeks after starting tenofovir alafenamide treatment. Liver FNAs were subjected to single-cell RNA sequencing and analysed using the Viral-Track method.

**Results:**

HBV was the only viral genome detected and was enriched within hepatocytes. The 5′ sequencing technology identified protein-specific HBV transcripts and showed that tenofovir alafenamide therapy specifically reduced pre-genomic RNA transcripts with little impact on HBsAg or HBx transcripts. Infected hepatocytes displayed unique gene signatures associated with an immunological response to viral infection.

**Conclusions:**

Longitudinal liver sampling, combined with single-cell RNA sequencing, captured the dynamic impact of antiviral therapy on the replication status of HBV and revealed host–pathogen interactions at the transcriptional level in infected hepatocytes. This sequencing-based approach is applicable to early-stage clinical studies, enabling mechanistic studies of immunopathology and the effect of novel therapeutic interventions.

**Impact and Implications:**

Infection-dependent transcriptional changes and the impact of antiviral therapy on viral replication can be measured in longitudinal human liver biopsies using single-cell RNA sequencing data.

## Highlights

• 5′ single-cell RNA sequencing of liver FNAs captures transcriptional start sites of major HBV proteins and pgRNA in hepatocytes.
• Detection of HBV transcripts allowed us to compare host transcriptional profiles between infected/uninfected hepatocytes.
• Longitudinal data from liver FNAs showed that treatment with TAF specifically reduced pgRNA in infected hepatocytes.

## Graphical abstract

**Figure:**
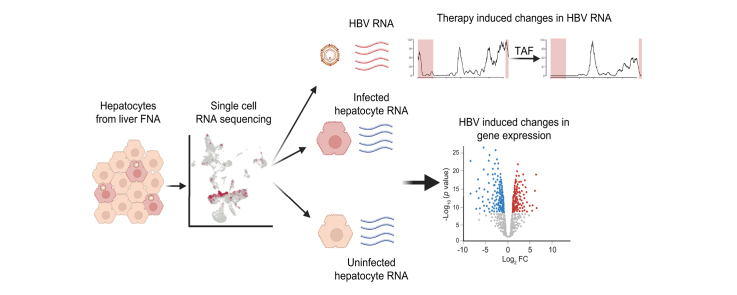

## Introduction

Chronic hepatitis B (CHB) is a primary cause of chronic liver disease, cirrhosis, and hepatocellular carcinoma and represents a major public health issue worldwide. More than 290 million people exhibit detectable levels of HBsAg and are living with CHB. Complications of chronic infection lead to the death of almost 1 million individuals with CHB each year.^1^ Because of this disease burden, intense effort is ongoing to identify new therapeutics that can cure CHB. However, these efforts have yet to improve cure rates in patients with CHB and require a better mechanistic understanding in the liver to refine therapeutic targets and identify viral and immunological biomarkers of control.[2], [3], [4]

Common human infections such as influenza have robust animal models that can be naturally infected to investigate host–pathogen interactions. In contrast, HBV infects only humans and chimpanzees. Therefore, patients now represent the only opportunity to study host–pathogen interactions that evolve over decades of chronic infection. Until recently, measuring host–pathogen interactions in the chronically infected human liver was hindered by limitations to elective, research-based access to liver tissue to capture key transitions in host–virus interactions and single-cell resolution of that response. Liver fine-needle aspirates (FNAs) have emerged as a method to electively sample the liver to capture dynamic changes induced by therapeutic intervention.[5], [6], [7], [8], [9] However, liver FNAs have primarily been used only to assess the activation status of intrahepatic immunity. The utility of liver FNAs to investigate the transcriptional response to infection in hepatocytes, or dynamic changes in HBV replication in hepatocytes induced by treatment, has not been demonstrated. Therefore, we are missing fundamental information related to the physiological status of infected *vs*. uninfected hepatocytes in patients with CHB and their innate response to HBV replication and treatment *in vivo*.

Viral-Track analysis has demonstrated the ability to extract viral genomes from single-cell RNA sequencing (scRNAseq) data.^10^ Because viral sequences contain the same unique barcode as the cells they were detected in, this can be used to specifically identify cells containing viral RNA. We hypothesised that using the Viral-Track method on scRNAseq data obtained from longitudinal liver FNAs of patients with CHB starting antiviral therapy would allow us to assess the therapeutic effect on HBV transcriptional activity. To test this hypothesis, we analysed scRNAseq data collected from liver FNAs of patients with CHB starting tenofovir alafenamide (TAF) therapy. Our data show that scRNAseq based on 5′ counting chemistry, combined with the Viral-Track analysis, enabled identification of individual HBV transcripts within infected hepatocytes, which changed in their distribution after starting TAF therapy. HBV transcripts were also found within macrophages. The presence of HBV transcripts allowed us to compare transcriptional differences between infected and uninfected hepatocytes and macrophages ± HBV transcripts. These data demonstrate that scRNAseq of liver FNAs can be used to simultaneously assess the effect of HBV RNA on immunological populations and infection status within hepatocytes. Therefore, longitudinal liver FNAs present an opportunity to gain a wholistic picture of the liver during treatment intervention.

## Patients and methods

### Ethical statement

This investigator-initiated clinical study (NCT04070079) was approved by the University Health Network Research Ethics Board (CAPCR ID: 18-5748), and written informed consent was obtained from all participants.

### Patient details

Five patients were included in this study. Inclusion criteria were chronic hepatitis B (HBsAg(+) ≥6 months); age >18 years; elevated alanine aminotransferase (ALT) levels, defined as >19 IU/L for females and >30 IU/L for males (with the upper limit of normal defined as >25 IU/L for females and >35 IU/L for males); HBV DNA >10,000 IU/ml for HBeAg(+) and >1,000 IU/ml for HBeAg(−) patients; and adequate contraception. Detailed clinical data have been reported.^9^ All patients had elevated ALT levels above the upper limit of normal at baseline. Exclusion criteria were antiviral or pegylated-interferon treatment in the previous 6 months; immunosuppressive treatment in the previous 6 months; treatment with an investigational drug in the previous 3 months; history of decompensated liver cirrhosis; liver transplantation; coinfection with HCV, HDV, or HIV; other significant liver disease (such as alcoholic or drug-related liver disease, autoimmune hepatitis, haemochromatosis, Wilson’s disease, or α1 antitrypsin deficiency); estimated glomerular filtration <50 ml/min/1.73 m^2^ or significant renal disease; α-foetoprotein >50 ng/ml; pregnancy or breastfeeding; other significant medical illness that might interfere with the study (*e.g.* immunodeficiency syndromes or malignancies); and substance abuse.

### Study design

This was an investigator-initiated, open-label phase IV study at the Toronto Centre for Liver Disease, Canada. Patients started therapy with 25 mg/day TAF for the entire study duration of 48 weeks and were offered to continue therapy after the end of the study. FNA samples were collected at baseline, Week 12, and Week 24 after staring TAF therapy.

### Liver FNA collection, processing, and sequencing

These methods were previously published.^9^ Briefly, liver FNAs were collected after ultrasound guidance by a hepatologist using 25-gauge spinal needles for puncture and aspiration of cells. A total of four liver FNA passes were collected from each patient at each time point. A small fraction of each pass was used to collect optical density to obtain a quantitative measure of the blood content.^9^ For analysis, we used the one or two passes with the lowest blood content from the respective time point and patient. Red blood cells were removed by 5-min incubation with Red Blood Cell Lysis Buffer (BioLegend, San Diego, CA, USA).

For scRNAseq, samples were prepared as outlined by the 10x Genomics Single Cell 5′ Reagent Kit (Pleasanton, CA, USA) user guide with a capture target of 3,000 cells. 5′ cDNA libraries were prepared as outlined by the 10x Genomics Single Cell 5′ Reagent Kit user guide, with modifications to the PCR cycles based on the calculated cDNA library input. Sequencing libraries were generated with unique sample indices for each sample and quantified.

The molarity of each library was calculated based on library size as measured by the Bioanalyzer (Agilent Technologies, Santa Clara, CA, USA) and quantitative PCR (qPCR) amplification data. Samples were pooled and adjusted to 10 nM and then diluted to 2 nM. Each 2 nM pool was denatured. Library pools were further diluted to a final loading concentration of 14 pM. Afterwards, 150 μl was loaded into each well of an eight-well strip tube and loaded onto a cBot (Illumina, San Diego, CA, USA) for cluster generation. Samples were sequenced on the HiSeq 2500 (Illumina) system.

### Cell annotation and viral identification

Quantitative analyses of transcriptomic data were performed using R package Seurat (version 4).[11], [12], [13], [14] Cell clustering was performed by using 2,000 variable genes, and 30 principal components (PCs) were included for dimensionality reduction. Shared nearest neighbor (SNN) graph was built with a parameter K equal to 20, and the Louvain method was used for clustering (resolution, 0.8). Data were visualised using uniform manifold approximation and projection implemented by the Seurat package.^15^ The viral read filtering and alignment were performed using Viral-Track^10^ using the viruSITE database (release 2020.3), containing 12,163 genome sequences from 9,297 viruses.^16^ Differential gene expression analysis was performed using MAST.^17^ Pathway analysis for hallmark gene signatures from MSigDB^18^ was done with the single-sample gene set-enrichment analysis method using R package escape.^19^

HBV genotyping from liver scRNAseq data was performed using HBVseq^20^ and the NCBI Genotyping tool.^21^ The viral contigs from Viral-Track analysis were combined for each patient and used as inputs for genotyping pipelines. Genotyping results were confirmed with Sanger sequencing. Briefly, 200 μl of baseline serum was used to extract viral DNA using the DNeasy Blood & Tissue Kit (QIAGEN, Canada) according to the manufacturer’s instructions. HBV surface gene was amplified with 5′-TCACCATATTCTTGGGAACAAGA-3′ and 5′-CGAACCACTGAACAAATGGC-3′ as forward and reverse primers, respectively, and the PCR product was sequenced using 5′-TGGGAACAAGAGCTACAGCATGG-3′ as a sequencing primer. HBV genotype and percent homology were determined using the NCBI HBV genotyping and nBLAST homology tools.

## Results

### Viral-Track analysis identified HBV transcripts at all three liver FNA time points

**Fig. 1:**
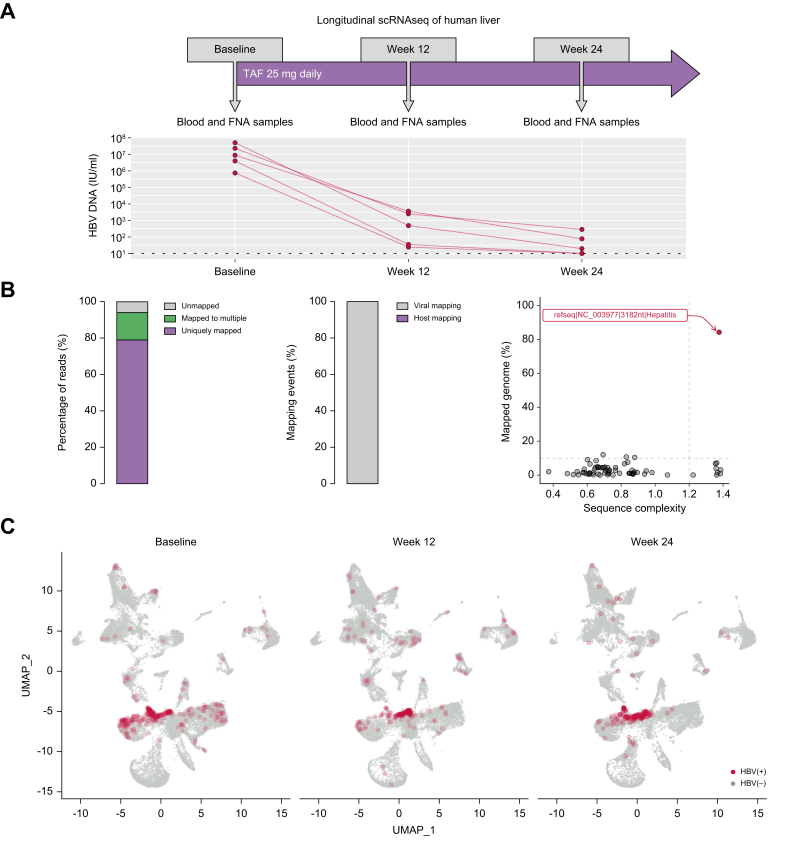
Identification of HBV transcripts in longitudinal scRNAseq samples. (A) Study design and HBV viral load decline after starting TAF therapy. (B) Identification of HBV transcripts and representative quality control metrics: (left) overall mapping quality of sequencing reads including both host and viral transcripts; (middle) proportion of mapping events that matched viral genomes after removing human sequences; and (right) the percentage of mapped viral contigs and the sequence complexity based on entropy calculation. (C) Infection map showing HBV transcripts in liver FNAs at each time point for all patients in the study. FNA, fine-needle aspirate; scRNAseq, single-cell RNA sequencing; TAF, tenofovir alafenamide.

The five patients enrolled for this study had active hepatitis with elevated ALT and HBV DNA above 10^5^ IU/ml, and two patients were HBeAg(+) (Table S1). Liver FNAs were collected at baseline, and 12 and 24 weeks after starting TAF, which decreased HBV replication (Fig. 1A). FNAs were all well tolerated and performed in the ambulant clinic with 30-min observation afterwards. Longitudinal liver FNAs from each patient were subjected to 5′ scRNAseq. We applied the Viral-Track analysis to the data and identified HBV transcripts. The majority of detected reads were uniquely mapped to the reference genome sequences, and after removing human host sequences, the remaining reads were mapped nearly 100% specific for the viral genome database. Only the HBV was identified as a true hit based on two quality metrics: the percentage of mapped viral genome segments and the sequence complexity (Fig. 1B). We generated an infection map and found HBV viral transcripts at each time point, which appeared to cluster, suggesting enrichment in specific cell types (Fig. 1C). These data demonstrated that we could capture HBV transcripts using the liver FNA approach at each time point, potentially allowing us to investigate the impact of HBV infection and TAF therapy.

### 5′ scRNAseq mapped the start sites of individual HBV protein transcripts

**Fig. 2:**
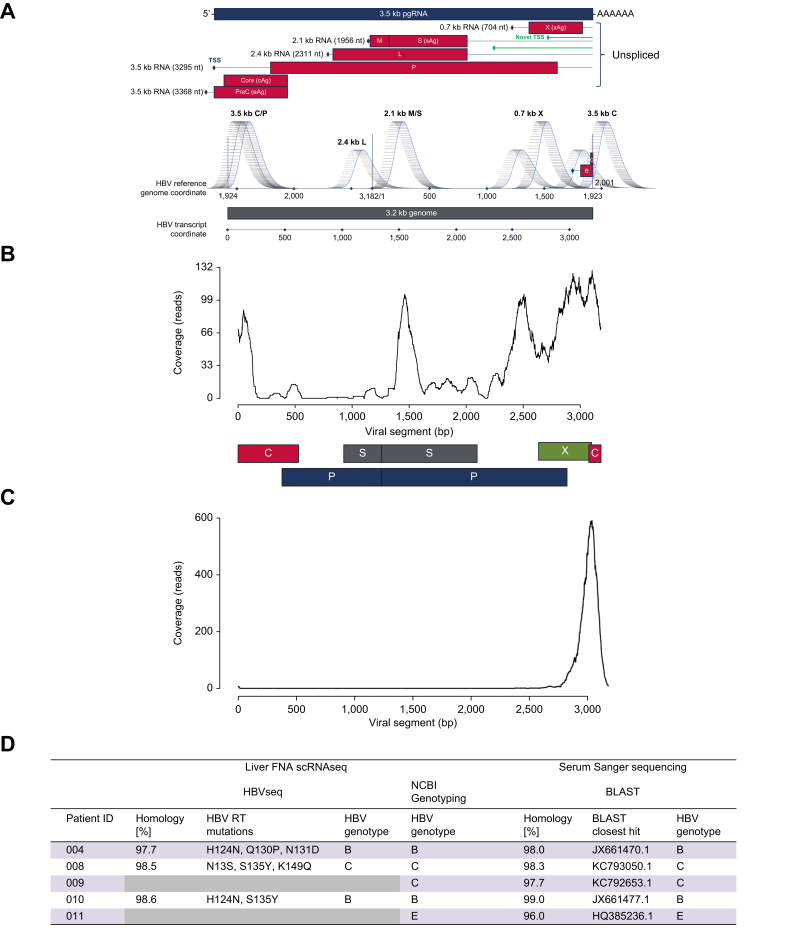
Identification of individual HBV transcripts with 5′ scRNAseq and genotype information. (A) Schematic representation of the linearised HBV genome with peaks indicating sequenced regions for each HBV transcript. (B) Representative viral genome coverage map of 5′ scRNAseq data from Viral-Track analysis. (C) Representative Viral-Track result from 3′ scRNAseq data. (D) HBV genotypes identified in scRNAseq data and by conventional Sanger sequencing. FNA, fine-needle aspirate; pgRNA, pre-genomic RNA; scRNAseq, single-cell RNA sequencing; RT, reverse transcriptase; TSS, transcription start site.

Knowing that HBV transcripts were detectable within our dataset, we next determined whether the 5′ sequencing approach would allow us to identify individual HBV transcripts. We linearised the HBV genome by setting the coordinates for viral transcripts to start immediately after the viral poly(A) signal (TATAAA at nucleotides 1918–1923 on HBV strain ayw genome; Fig. 2A and Fig. S1) and annotated with recent transcription start site mapping information.^22^^,^^23^ With these coordinates, the 3.5-kb pre-genomic RNA (pgRNA) starts at the far left on the circular genome, the HBsAg transcripts are located in the centre of the genome, and the HBx transcription start sites are located at the right end of the genome. The sequenced regions predicted based on library fragment size and sequencing read length are indicated by bell-shaped peaks below the linear HBV genome map (Fig. 2A). Based on this convention, we observed strong peaks near the pgRNA, HBsAg, and HBx start sites (Fig. 2B). As a comparison, we performed the Viral-Track analysis on samples that were analysed using 3′ counting chemistry for scRNAseq. Using the same transcript coordinate system for 3′ scRNAseq, we observed only a single peak at the 3′ location for HBV transcripts (Fig. 2C), consistent with the original reported data from Viral-Track analysis.^10^ This is consistent with the HBV genome structure having one common 3′ termination site for all major HBV transcripts.

Given the ability to sequence individual HBV transcripts, we tested whether the sequence data were of sufficient quality to determine HBV genotype within each patient. In three of the five patients, we were able to resolve the HBV genotype by both HBVseq and the NCBI Genotyping tool, which was validated by traditional sequencing-based genotyping using the serum of each patient at baseline (Fig. 2D). For two patients, HBVseq failed to produce genotypes because of poor coverage for viral reverse transcriptase mutations. However, the NCBI Genotyping tool yielded HBV genotypes matching Sanger sequencing results for these two patients. These data demonstrate the potential utility of 5′ scRNAseq in the Viral-Track analysis, allowing us to genotype the virus and investigate the impact of therapy on individual HBV RNA transcripts.

### Infection map localises HBV transcript to hepatocytes

**Fig. 3:**
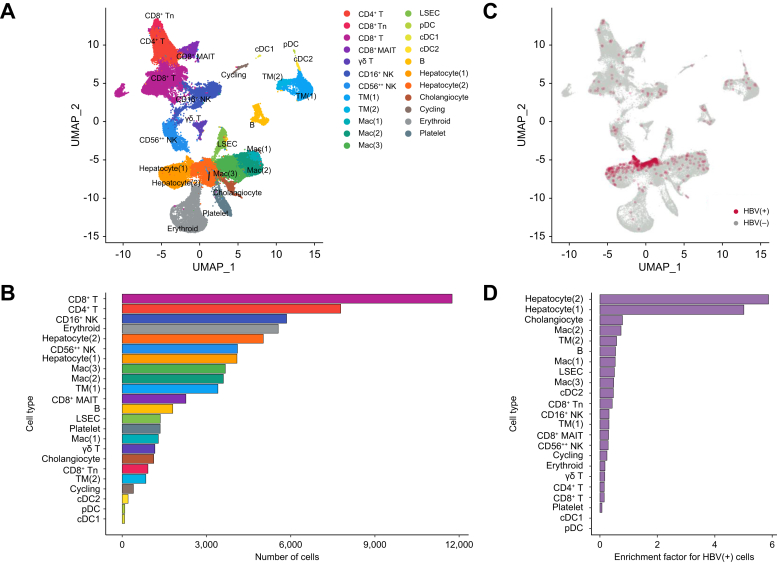
Localisation of HBV within hepatocytes. (A) Cell annotation of liver FNA 5′ scRNAseq dataset. (B) Number of cells in each cluster. (C) Infection map projected onto cell annotation UMAP. (D) HBV transcript enrichment factor in each cell cluster defined as the ratio of HBV(+) cell frequency within each cluster relative to the frequency of HBV(+) cells in the total cell populations. CD56++ NK, CD56^bright^ NK cell; cDC, conventional dendritic cell; FNA, fine-needle aspirate; LSEC, liver sinusoidal endothelial cell; Mac, macrophage; MAIT, mucosal-associated invariant T cell; NK, natural killer; pDC, plasmacytoid dendritic cell; scRNAseq, single-cell RNA sequencing; TM, tissue monocyte; Tn, naïve T cell.

Having established the specificity of the data, and the potential to analyse individual HBV transcripts, we next wanted to confirm that these transcripts were primarily localised within hepatocytes. It was possible that HBV transcripts could come from residual serum, or lysed hepatocytes during cell capture, and spread randomly across the cellular dataset. To generate cell clusters to overlay with the infection map, we integrated data from all samples and identified major cell types in uniform manifold approximation and projection plots using canonical marker genes (Fig. 3A and Fig. S2). To include as many hepatocytes and parenchymal cells as possible in this analysis, cell quality parameters were set to include cells with up to 50% mitochondrial gene content with a minimum of 20 genes/cell and 50 unique molecular identifiers per cell. Using these parameters, we could readily identify the major immune cell populations, including T cells, B cells, natural killer cells, and macrophages (Fig. 3A). In addition, we found significant numbers of hepatocytes but much lower numbers of liver sinusoidal endothelial cells and cholangiocytes, which were not captured in all patients at all time points (Fig. 3B). These data show that although parenchymal cells were underrepresented in liver FNAs, significant numbers of hepatocytes were captured for each patient at each time point, albeit with some variability (Fig. S3).

When the infection map was overlaid onto the annotated cell map, it was visually apparent that most HBV transcripts were observed within the hepatocyte clusters (Fig. 3C). To quantify enrichment of HBV transcripts for each cell type, we calculated the ratio of HBV(+) cell frequency within each cluster relative to the frequency of infected cells in the total cell populations (Fig. 3D). Using this method, we found that two hepatocyte clusters showed the greatest enrichment of viral transcripts, followed by cholangiocytes and macrophages. Owing to the small number of cholangiocytes and the low number of genes detected per cholangiocyte, it is difficult to confirm the viral infection of cholangiocytes. However, the data suggest that a small fraction of liver macrophages are positive for HBV transcripts, which could come from phagocytosed viral particles or dying infected hepatocytes. Despite a scattering of HBV sequences found in other lymphocyte clusters, the very low viral enrichment factors in those lymphocyte clusters suggested that these were non-specific signals. These data further validate the Viral-Track analysis, primarily localising HBV transcripts to hepatocytes, the reservoir of infection, and macrophages, which are known to engulf dead hepatocytes and potentially HBV virions.^24^^,^^25^

### Transcriptional differences between HBV(+) or HBV(−) hepatocytes and macrophages

**Fig. 4:**
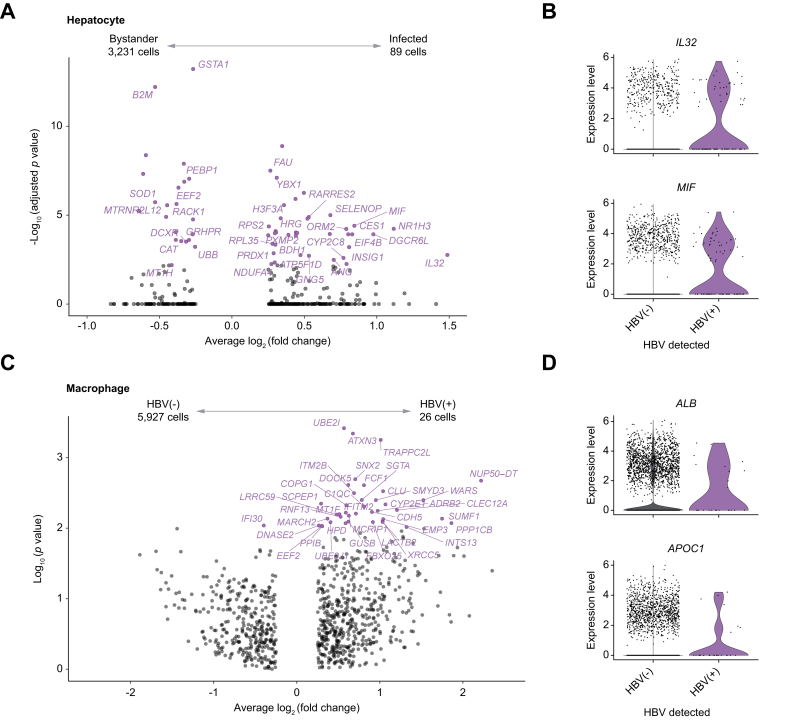
Transcriptional profile in infected *vs*. noninfected hepatocytes and macrophages ± HBV transcripts. (A) Volcano plots demonstrating significant DEGs in infected hepatocytes. (B) Violin plots confirming significant upregulation of inflammatory genes in infected hepatocytes. (C) Volcano plots of macrophages ± HBV transcripts. (D) Violin plots showing significant increases of hepatocyte genes in HBV(+) macrophages. DEG analysis was performed using MAST.^17^ ALB, albumin; APOC1, apolipoprotein C–I; DEG, differentially expressed gene; MIF, macrophage migration inhibitory factor.

Now that we could identify cells that do or do not contain HBV transcripts, we used this as a variable to compare transcriptional profiles of infected *vs*. noninfected hepatocytes and macrophages ± HBV transcripts. To assess biological changes between infected and uninfected hepatocytes, we performed differential gene expression on hepatocytes that passed a more stringent quality cut-off (<25% mitochondrial gene content). Increased stringency largely excluded the Hepatocyte(2) cluster from this analysis because of their higher mitochondrial gene content. In infected hepatocytes, we found upregulation of some immune genes, including macrophage migration inhibitory factor (MIF) and IL-32, compared with that in uninfected hepatocytes (Fig. 4A). The differential expression between infected and noninfected hepatocytes was highly significant for these two genes: adjusted *p* values of 3.87 × 10^-5^ (MIF) and 0.00174 (IL-32) (Fig. 4B). We then compared macrophages, ± HBV transcripts, and found minimal changes (upregulation or downregulation) in immune response-related genes in HBV(+) macrophages, which was confirmed by pathway analysis (Fig. 4C and Fig. S4). However, we noted that HBV(+) macrophages contained a higher proportion of cells with albumin and apolipoprotein C–I transcripts, suggesting that these cells likely acquire HBV transcripts through phagocytosis of HBV-infected hepatocytes (Fig. 4D). This interpretation is supported by the observation that multiple HBV transcription start sites could be detected in macrophages rather than just pgRNA that may come from RNA containing capsids (Fig. S5). The expected multiplet rate based on the loaded cell counts is approximately 3%. Therefore, it is unlikely that the HBV(+) macrophages result from doublet formation during droplet capture. These data demonstrate that scRNAseq data can be used to discriminate infected *vs*. noninfected hepatocytes and identified immune cells (macrophages), known to phagocytose dying hepatocytes, as HBV transcript positive.

### Viral-Track analysis captures the therapeutic impact of TAF treatment on individual HBV transcripts

**Fig. 5:**
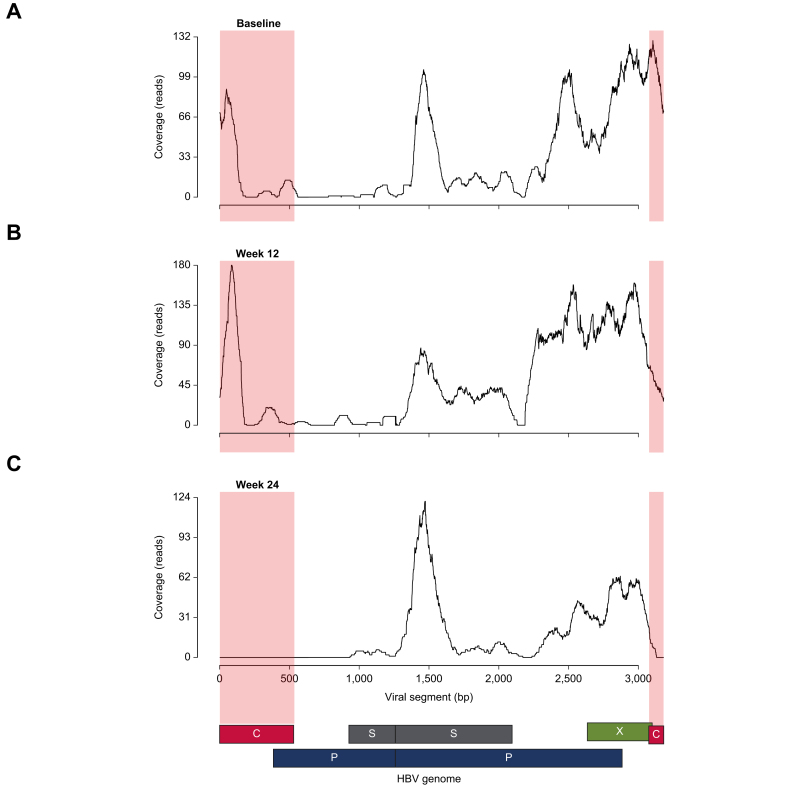
Disappearance of pgRNA peak after 24 weeks of TAF therapy. HBV RNA transcript expression patterns at (A) baseline, (B) after 12 weeks of therapy, and (C) after 24 weeks of therapy, showing patient samples with the highest genome coverage at each time point. pgRNA, pre-genomic RNA; TAF, tenofovir alafenamide.

The other goal of our study was to determine whether 5′ scRNAseq and Viral-Track analysis was sensitive enough to measure changes in HBV transcript distribution after therapeutic intervention. To do this, we compared the Viral-Track trace at each time point within patients after starting TAF therapy. At baseline, we measured peaks for all major HBV transcripts, namely, pgRNA, HBs, and HBx (Fig. 5A). A similar trace was observed after 12 weeks of TAF (Fig. 5B). However, after 24 weeks of TAF therapy, the pgRNA transcripts had become undetectable, whereas HBs and HBx transcripts persisted (Fig. 5C). These data indicate a specific loss of detection of the pgRNA after 24 weeks of TAF therapy. We observed similar profiles for the other patients, although HBV transcripts were not detectable at all time points (Fig S6). These data demonstrate the utility of liver FNAs analysed by 5′ scRNAseq to measure dynamic changes in specific HBV transcripts after treatment intervention.

## Discussion

scRNAseq has provided unprecedented detail into the immunological response during chronic HBV infection. However, the analysis has been restricted to associations between virological/clinical parameters with immune status.^9^^,^^26^^,^^27^ Our analytical approach, using Viral-Track on a dataset where immunological responses were analysed,^9^ will allow us to bring these components together, comparing the effects of therapy on infected and uninfected hepatocytes and macrophages ± HBV transcripts. In our proof-of-concept study, we showed that HBV-infected hepatocytes upregulated immune genes, whereas HBV-transcript-positive macrophages showed no additional inflammatory response compared with HBV-negative macrophages. Not only could we compare infected *vs*. noninfected cells, but we also measured dynamic changes in individual HBV transcripts following therapeutic intervention. This opens greater potential to use scRNAseq technologies in clinical studies to simultaneously monitor the immune response to treatment intervention and the antiviral effects of therapy.

The value of using the 5′ sequencing technology was immediately evident in the Viral-Track analysis. Because all HBV transcripts terminate at a single site, 3′ sequencing showed only a single peak at the expected location in the viral genome. In contrast, we detected individual transcripts for HBc/HBe, HBs, and HBx proteins. Not only were individual transcripts detectable, but their distribution changed with therapy, where the pgRNA transcripts decreased in patients with CHB after 24 weeks of therapy. This is consistent with previously published data showing decreased pgRNA detection after nucleoside analogue treatment in individual hepatocytes analysed by laser-capture microdissection, suggesting downregulation of transcription from covalently closed circular DNA during nucleoside analogue treatment.^28^ Because TAF blocks reverse transcription, and not transcription of HBV RNA, we speculate that the specific loss of detection of pgRNA may be related to the chain-terminating nature of TAF and RNase H activity of HBV polymerase, resulting in the loss of poly(A) tails interfering with the 5′ sequencing chemistry. In addition, the pgRNA transcription start sites are located upstream of the DR1 locus, the common integration junction on the HBV genome. Therefore, our data are consistent with the notion that, after integration into the host genome, integrated HBV DNA is unable to produce pgRNA transcripts because their open reading frames are separated from the promoters, whereas HBs and HBx open reading frame expression remains intact.[29], [30], [31] The ability to monitor individual HBV transcripts over time in liver FNAs opens opportunities to measure the activity of direct-acting antivirals, particularly siRNAs and anti-sense oligonucleotides, and the impact of immunomodulatory therapies on HBV transcripts.

The detection of HBV transcripts provided the first opportunity to compare the impact of HBV infection on the immunological status of infected *vs*. noninfected hepatocytes *in vivo*, in the livers of patients with CHB. Although the differences were modest in this dataset, HBV-infected hepatocytes displayed upregulation of cytokines that can promote inflammation. In infected hepatocytes, the upregulation of MIF, in particular, has the potential to promote liver damage by enhancing pathogenic inflammation.[32], [33], [34], [35] It is likely that the differences between infected and uninfected hepatocytes were relatively small in our analysis because the data were derived from patients with chronic active hepatitis. Therefore, the inflamed liver microenvironment may have already increased immune-related genes in uninfected hepatocytes, and macrophages, reducing our ability to resolve differences between the two populations. In a setting of inactive disease, where ALT is normal, the differences between infected and uninfected hepatocytes may become more apparent.

Detecting HBV(+) macrophages in the dataset was anticipated. We have demonstrated that monocytes can internalise HBV antigens, and others have shown that macrophages can do the same.^25^^,^^36^^,^^37^ We anticipated two potential profiles of HBV transcripts within macrophages: one dominated by pgRNA internalised from the extracellular environment in RNA-containing viral particles or the full profile of transcripts that would result from phagocytosing dying infected hepatocytes. What we found was the latter (not shown), multiple HBV transcripts within the macrophage population along with enrichment of hepatocyte-specific transcripts of albumin and apolipoprotein C–I in the HBV(+) population. These data argue strongly that macrophages phagocytosed dying infected hepatocytes. We cannot be certain that the viral transcripts present in macrophages are caused by phagocytosis of infected hepatocytes rather than by hepatocyte–macrophage doublets captured during the loading process. However, these cells passed quality control filtering to remove doublets based on unique molecular identifiers and gene counts. Phagocytosis of dying hepatocytes has been described in animal models and provides a route for HBV RNA or hepatocyte-targeting drugs to enter macrophages,^24^ which have the potential to trigger an inflammatory response.^36^^,^^38^^,^^39^

It is important to note that changes in hepatocyte and macrophage gene expression were assessed in relatively few HBV(+) cells because of increased stringency used for the differential comparison. This effort was made to provide robust biological data in the healthiest hepatocytes and was confirmed by highly significantly different expression of MIF and IL-32 in the violin plots. Because hepatocytes have also been detected in liver FNAs by flow cytometry,^7^ we are confident that optimised processing of the FNAs will yield more hepatocytes. Alternatively, single-nuclei sequencing is superior to single-cell sequencing for liver parenchymal cells and may further improve hepatocyte capture for analysis.^27^

Overall, this proof-of-concept study expands the utility of scRNAseq on liver FNAs, offering an opportunity to truly measure host–pathogen interactions within the human liver. We identified transcriptional differences in infected hepatocytes and anticipate that the frequent longitudinal sampling permitted by the FNA procedure will be highly valuable for novel therapies to validate the mechanism of action in patient livers. This method, based on standard scRNAseq, can be applied to other viral diseases (*e.g.* influenza and COVID-19) and library preparation methods,^10^ and the bioinformatics pipeline can be applied to previously obtained data from infected tissues where viral transcripts were not sought after. Targeted-enrichment for non-polyadenylated viral RNA species can extend this method to different types of viruses, as recently demonstrated in an animal model of reovirus-induced myocarditis.^40^ Lastly, although scRNAseq provides the added benefit of cellular transcriptional responses, the ability to detect transcripts in the 10x Genomics technology suggests that standard protocols, such as quantitative PCR, could be used to specifically assess viral replication in FNAs without the high costs associated with single-cell technologies.

## Financial support

This study was funded by an investigator-initiated grant by 10.13039/100005564Gilead Sciences. Adam J. Gehring received funding from the Canada Foundation for Innovation John R. Evans Leadership Fund and 10.13039/100017784Gilead Research Scholars, North America Grant.

## Authors’ contributions

Conceived the study: AJG, SCK, JJW. Performed bioinformatic data analysis for the paper: SCK, YG, MAZ, JDSV, SN. Wrote the manuscript: SCK, AJG. Reviewed study data, provided input on project direction, and revised the manuscript: SF, PM, JJF, HLAJ.

## Data availability statement

Viral sequence data are available on the NCBI Gene Expression Omnibus (GEO), accession number GSE234015.

## Conflicts of interest

SK, JJW, and PM are employees of Gilead Sciences. AJG has received research funding from Gilead Sciences, Janssen Pharmaceuticals, and GSK, and performs consulting/scientific advisory services for Janssen Pharmaceuticals, Roche, GSK, Vir Biotech, Virion Therapeutics, and Bluejay Therapeutics. HLAJ received research funding from AbbVie, Gilead Sciences, GlaxoSmithKline, Janssen, Roche, and Vir Biotechnology Inc., and performs consulting/scientific advisory services for Aligos, Antios, Arbutus, Eiger, Gilead Sciences, GlaxoSmithKline, Janssen, Merck, Roche, VBI Vaccines, Vir Biotechnology Inc., and Viroclinics. JJF received research funding from Abbvie, Gilead, GlaxoSmithKline, Janssen, Roche, Eiger, and Enanta, and performs consulting/scientific advisory services for Abbvie, Antios, Arbutus, Eiger, Enanta, Bluejay, GSK, Janssen, and Roche. SF receives speaking/teaching fees from Gilead Sciences and research funding from Gilead Sciences, and performs consulting/scientific advisory services for Gilead Sciences, Abbvie, Janssen, Spring Bank, Pfizer, and Novo-Nordisk.

Please refer to the accompanying ICMJE disclosure forms for further details.
