# Supplementary material for: Efficacy of antiviral therapy and host–virus interactions visualised using serial liver sampling with fine-needle aspirates
Source: JHEP Rep. 2023 Jun 19;5(9):100817. doi: 10.1016/j.jhepr.2023.100817 (PMC10432215; doi:10.1016/j.jhepr.2023.100817)
Supplement: Multimedia component 1 [file mmc1.pdf]

Efficacy of antiviral therapy and host-virus interactions visualized using serial liver sampling with fine-needle aspirates

Samuel C. Kim, Jeffrey J. Wallin, Yanal Ghosheh, Muhammad Atif Zahoor, Juan Diego Sanchez Vasquez, Shirin Nkongolo, Scott Fung, Patricia Mendez, Jordan J. Feld, Harry L.A. Janssen, Adam J. Gehring

Table of contents

Fig. S1 2
Fig. S2 3
Fig. S3 4
Fig. S4 5
Fig. S5 6
Fig. S6 7
Table S1 8

HBV transcripts and their expected coverage from 10x 5' chemistry

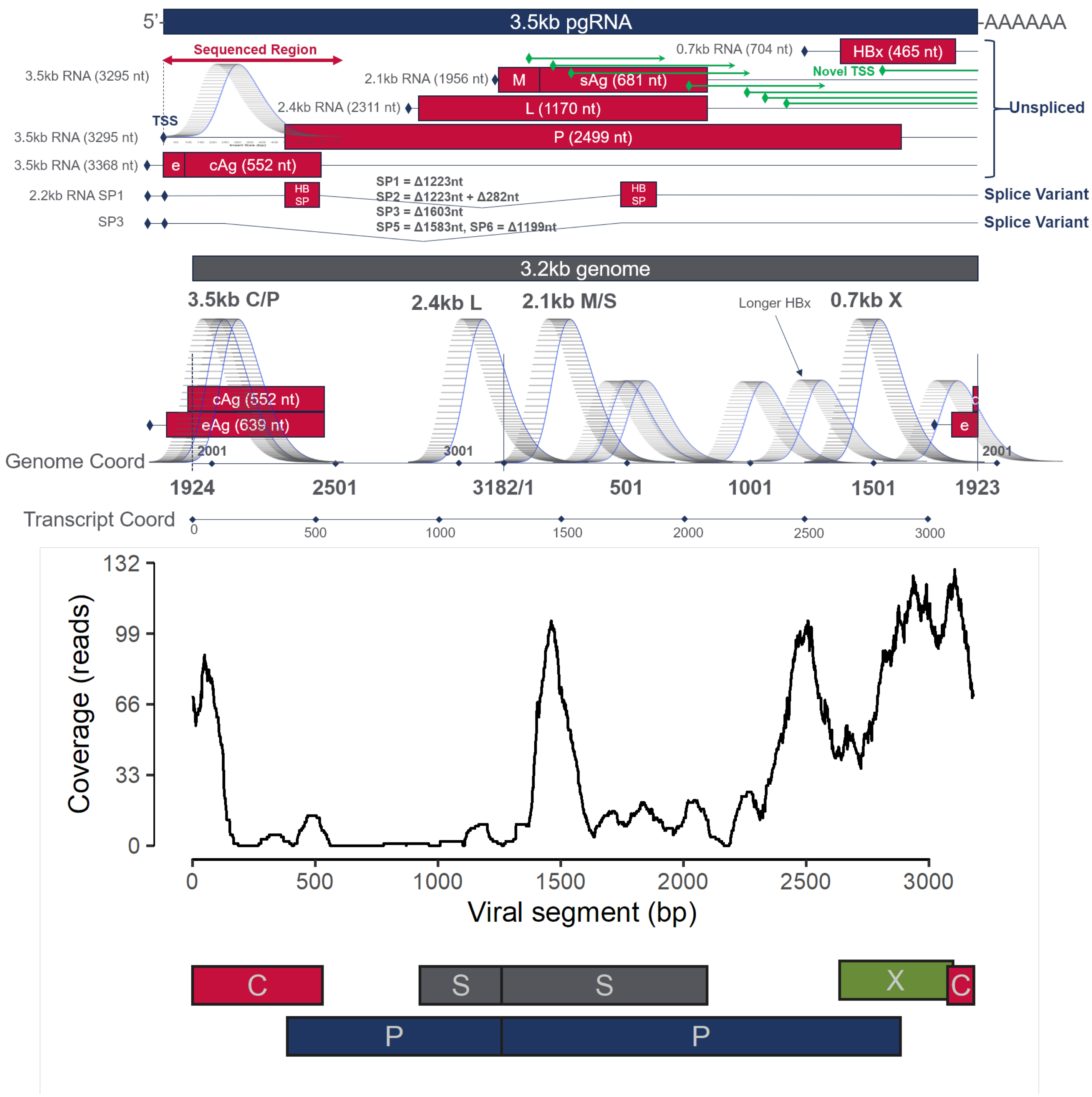

Fig. S1. Schematic representation of the HBV genome with peaks indicating sequenced regions for each HBV transcript including additional novel transcription start sites (TSS) and potential splice variants.

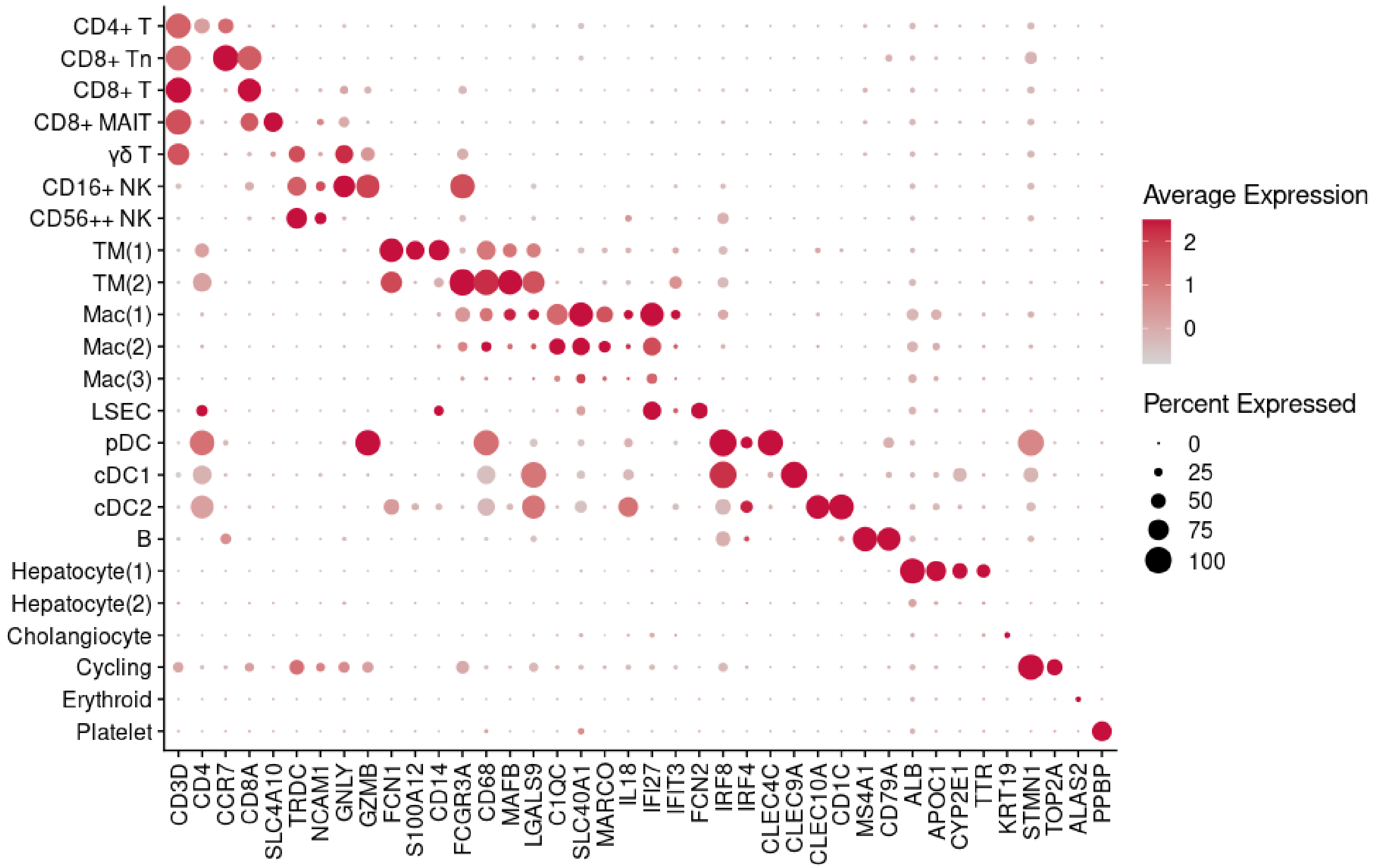

Fig. S2. Dot plot showing the average expression levels of curated marker genes used for cell type annotation.

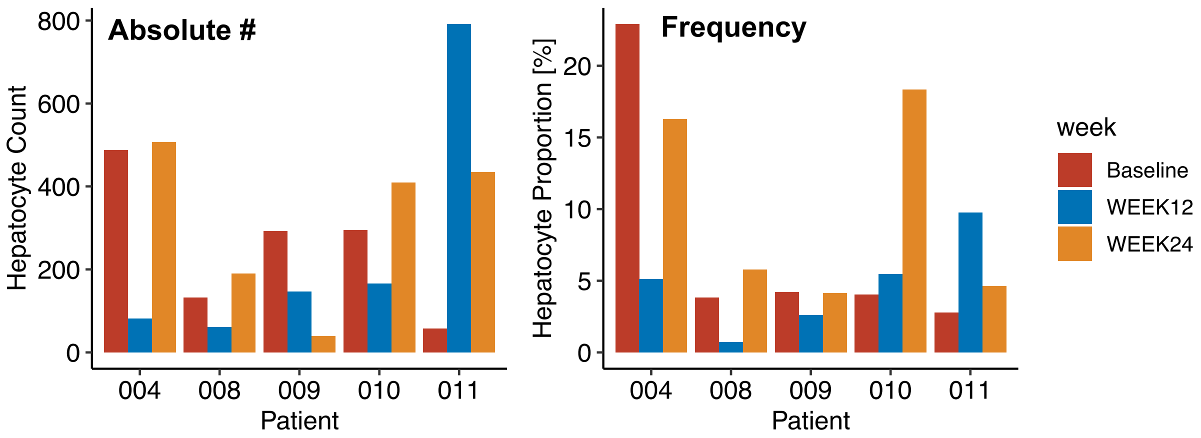

Fig. S3. Absolute hepatocyte count (left) and frequency (right) in each patient at each time point.

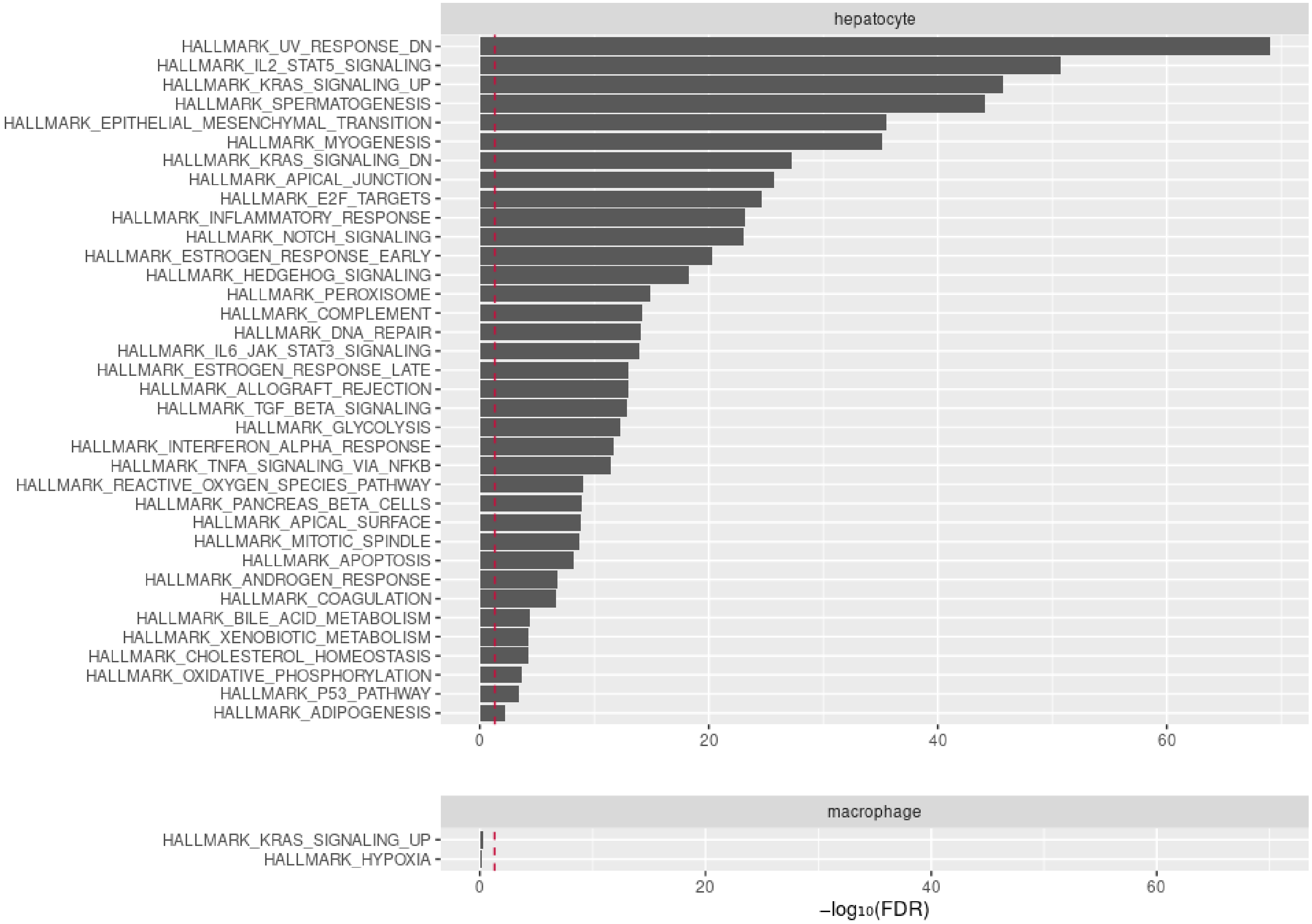

Fig. S4. Pathway analysis for differentially expressed genes in hepatocytes and macrophages with or without HBV transcripts. The red dotted line corresponds to 0.05 false discovery rate (FDR) value.

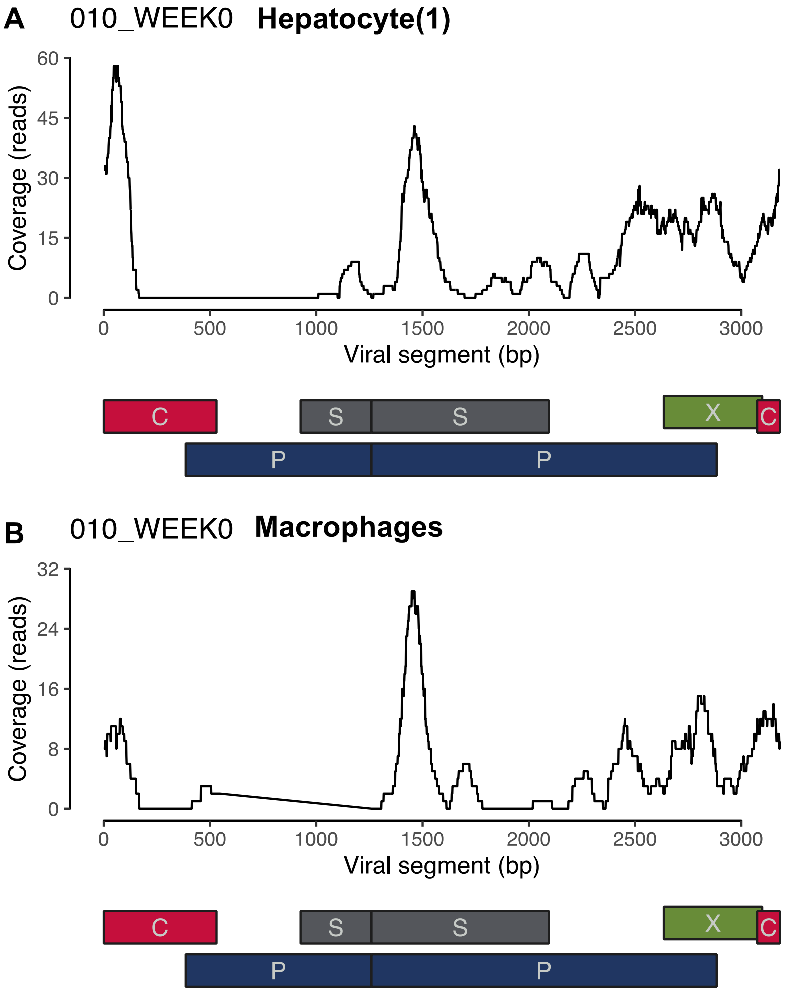

Fig. S5. HBV transcripts detected in macrophages. HBV transcription start site trace in A) Hepatocyte(1) and B) macrophages from the baseline sample of patient 10. Multiple transcription start sites in macrophages suggests internalization of dead, infected hepatocytes during active liver damage.

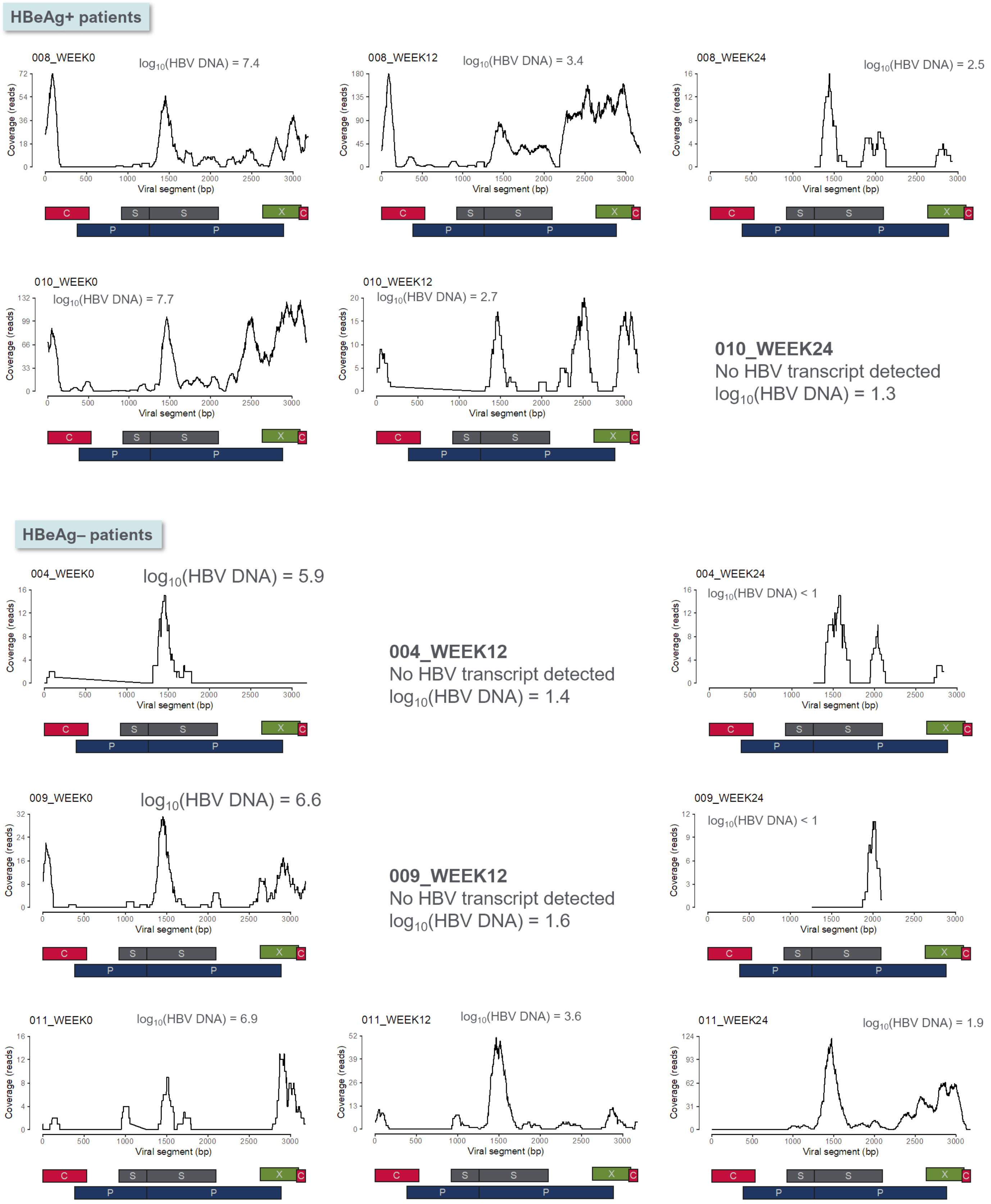

Fig. S6. Viral-Track traces from all time points from all 5 patients. Time points go left to right. Top two rows are HBeAg(+) patients and bottom three rows are HBeAg(–) patients.

Patient	Visit	HBeAg	HBV DNA [IU/mL]	ALT [IU/L]
004	Screening	–	745,000	92
	Baseline	–		102
	Week 12	–	24.3	30
	Week 24	–	< 10	21
008	Screening	+	23,100,000	36
	Baseline	+		40
	Week 12	+	2,510	36
	Week 24	+	285	34
009	Screening	–	3,990,000	581
	Baseline	–		468
	Week 12	–	35.1	24
	Week 24	–	< 10	30
010	Screening	+	50,400,000	299
	Baseline	+		326
	Week 12	+	487	110
	Week 24	+	19.6	19
011	Screening	–	8,810,000	186
	Baseline	–		126
	Week 12	–	3,640	42
	Week 24	–	77.0	27

Table S1. Clinical parameters from virological and clinical chemistry laboratory measurements.
